# The role of JMJD6/U2AF65/AR-V7 axis in castration-resistant prostate cancer progression

**DOI:** 10.1186/s12935-020-01739-1

**Published:** 2021-01-11

**Authors:** Dali Tong

**Affiliations:** Department of Urology, Daping Hospital, Army Medical University, Chongqing, People’s Republic of China

## Abstract

Castration-resistant prostate cancer (CRPC) remains prostate cancer research and treatment bottleneck. Abnormal androgen receptor (AR) activation still has a pivotal role in CRPC. Multiple mechanisms involve the process, of which overabundant AR-V7 mRNA splicing production is currently focused and increasingly studied. However, factually, there is no definite conclusion about regulation of AR-V7 mRNA splicing. Recently developed knowledge has demonstrated that JMJD6 and U2AF65 as a hopeful approach in mRNA splicing regulation. The authors propose a novel possible mechanism elucidating AR mRNA splicing for CRPC progression using dual-function enzyme JMJD6 and its induced JMJD6/U2AF65/AR-V7 axis. In this hypothesis JMJD6 introduces to AR promoter to demethylate H3R or H4R and promotes AR mRNA transcription via its demethylase activity and interaction with U2AF65. It is expected that JMJD6 could further effectively perform U2AF65 hydroxylation to achieve AR-V7 mRNA splicing via its hydroxylase activity.

## Background

Prostate cancer has become one of the most frequently diagnosed cancers of men in the western countries and the second-leading cause of male cancer death. Androgen-deprivation therapy (ADT), as the mainstay and milestone of PC treatment, remains standard selection for both advanced and metastatic prostate cancer patients. Unfortunately, although most patients are effective to ADT in early-stage, almost all of PC finally develop from androgen-dependent prostate cancer to castration-resistant prostate cancer (CRPC) after a median-survival time.

A panel of studies have confirmed that the androgen receptor (AR) still has a crucial role even in CRPC. Numerous mechanisms have been proposed to involve in the role of AR in CRPC progression, containing generation of truncated AR mutations and spliceosomes such as AR-V4, AR-V7, ARv567s, AR-V9 [1, 2], local synthesis of androgens enhancement, and upregulated AR transcription and translation. Thereinto, enhanced AR-V7 expression and activation are important factors promoting CRPC with no need of hormone stimuli, which is involved in multiple mechanisms including special AR-V7 mRNA splicing, decreased AR-V7 protein degradation and regulation of AR-V7 coactivators and corepressors. Up to now, special AR-V7 mRNA splicing in transcription level is one of recently discovered and mostly studied contents. A panel of evidence show that AR-V7 mRNA splicing, especially AR-V7 splicer regulation, is related to prostate cancer growth and development, resistance to castration and drug therapy [3]. mRNA splicing produces diverse transcripts from original pre-messenger RNA (pre-mRNA) by removing introns and promoting exons connection. However, the mechanisms leading to alternative AR-V7 splicing are not very clear.

U2AF65, as the important component of U2 mRNA spliceosome, used to assist U2 performing mRNA splicing by recognizing and binding with splicing identification sequence GU-AG. In aspects of molecular structure, U2AF65 is composed of an N-terminal arginine/serine-rich domain, two RNA-recognition motifs and C-terminal noncanonical RNA-recognition motifs [4, 5]. These domain conduct protein–protein and protein–RNA interactions. Moreover, mRNA splicing process often needs recruitment and coordination of a group of splicing factors at the sites of mRNA processing zone, such as U2AF35, PUF60, SPF45 and RBM39. Constitutive and alternative splicing performance also need help from short tryptophan-containing linear ligand motifs. Up to now, several U2AF65 spliceosomes have been discovered, including U2AF35–U2AF65 complex locating in the constitutive 3′ splice site [6], U2AF65-splicing factor SF1 binding to the Py-tract domain [7], alternative U2AF65–SPF45–RBM39 complex [8, 9] and U2AF65 heterodimers with different set of U2AF65-binding proteins. U2AF65 hydroxylation makes it more activated to regulate mRNA splicing process [10]. But how U2AF65 is activated to bind with mRNA is not largely known.

JMJD6 is upregulated and over-activated in a series of cancers including breast, lung, neuroglioma and melanoma [11]. Previous study found that JMJD6 is a member of histone demethylases responsible for demethylating H3R2 (histone H3 at arginine 2) and H4R3 (histone H4 at arginine 3). Interestingly, recent study found that JMJD6 is a Jumonji C domain-containing hydroxylase with enzyme activity for hydroxylating U2AF65 lysyl, indicating JMJD6 as a dual function enzyme either a histone arginine demethylase or hydroxylase. JMJD6 have been confirmed to bound efficiently to single-stranded RNA, not to single-stranded DNA, double-stranded RNA, or double-stranded DNA [12]. Moreover, JMJD6 could regulate splicing of constitutive splice reporter and bind with RNA derived from the reporter plasmid. JMJD6 exerts its splicing function by interacting with specific SR-related proteins in RNA-dependent or independent manner [13]. Recently, JMJD6 has been shown to interact with U2AF65, to catalyse hydroxylation of lysine residues in U2AF65 and to regulate alternative splicing. For example, JMJD6 interacts with the splicing factors U2AF65 and further binds to VEGF-receptor 1 mRNA to modulate its splicing [14].

## Presentation of the hypothesis

Altered splicing machinery based on RNA-binding protein splicing factor proline- and glutamine-rich results in a dysregulated AR splicing process and the expression of AR transcript variants including AR-V7. AR-V7 mRNA production requires the removal of alternative intron sequences via special RNA site recognition. The spliceosome excises the AR mRNA 3′ SS and ligates exon 3 with special exon 3B. Among these splicers, the recruitment of U2AF65 to the 3′ splice site of AR-V7 enhanced by ADT is of vital importance for increased expression of AR-V7 [10, 15]. Collectively, these evidences suggested that U2AF65 functions as the “commander” role of AR splicing machinery and prostate cancer development [15].

JMJD6, as dual-function regulators of both arginine demethylase and lysine hydroxylase, can regulate the alternative splicing through U2AF65 hydroxylation, suggesting the possibility of JMJD6 as splicing factors. Accordingly, the knock-down of JMJD6 in HEK-293T cells resulted in a significant change of the outcome for 14% of all alternative splicing events (n = 3710), and 74% of those were co-regulated with U2AF65 [16]. Combined with U2AF65 function as AR-V7 splicing, JMJD6 and U2AF65 appear to co-bind with the pre-mRNAs and thus co-regulate alternative AR-V7 splicing. It is noteworthy that JMJD6–U2AF65 interaction is JMJD6 enzymatic activity-dependent, but JMJD6-U2AF65 complex regulates AR-V7 splicing is in JMJD6 enzymatic activity- independent manner.

We hypothesized that JMJD6 can play the role of dual-functional enzyme in initiating AR mRNA transcription activation by demethylating histone in the manner of its demethylase activity and further promoting alternative AR-V7 mRNA splicing by hydroxylating and activating U2AF65 in the manner of its hydrolase activity. Upregulated JMJD6 usually progressively induce AR mRNA transcription, U2AF65 activation and ultimately special AR-V7 mRNA splicing. Therefore, the JMJD6/U2AF65/AR-V7 axis was established and it plays important regulatory roles in CRPC progression. We evaluated the current evidence supporting this hypothesis in the following part.

## Evaluation of the hypothesis

**Fig. 1:**
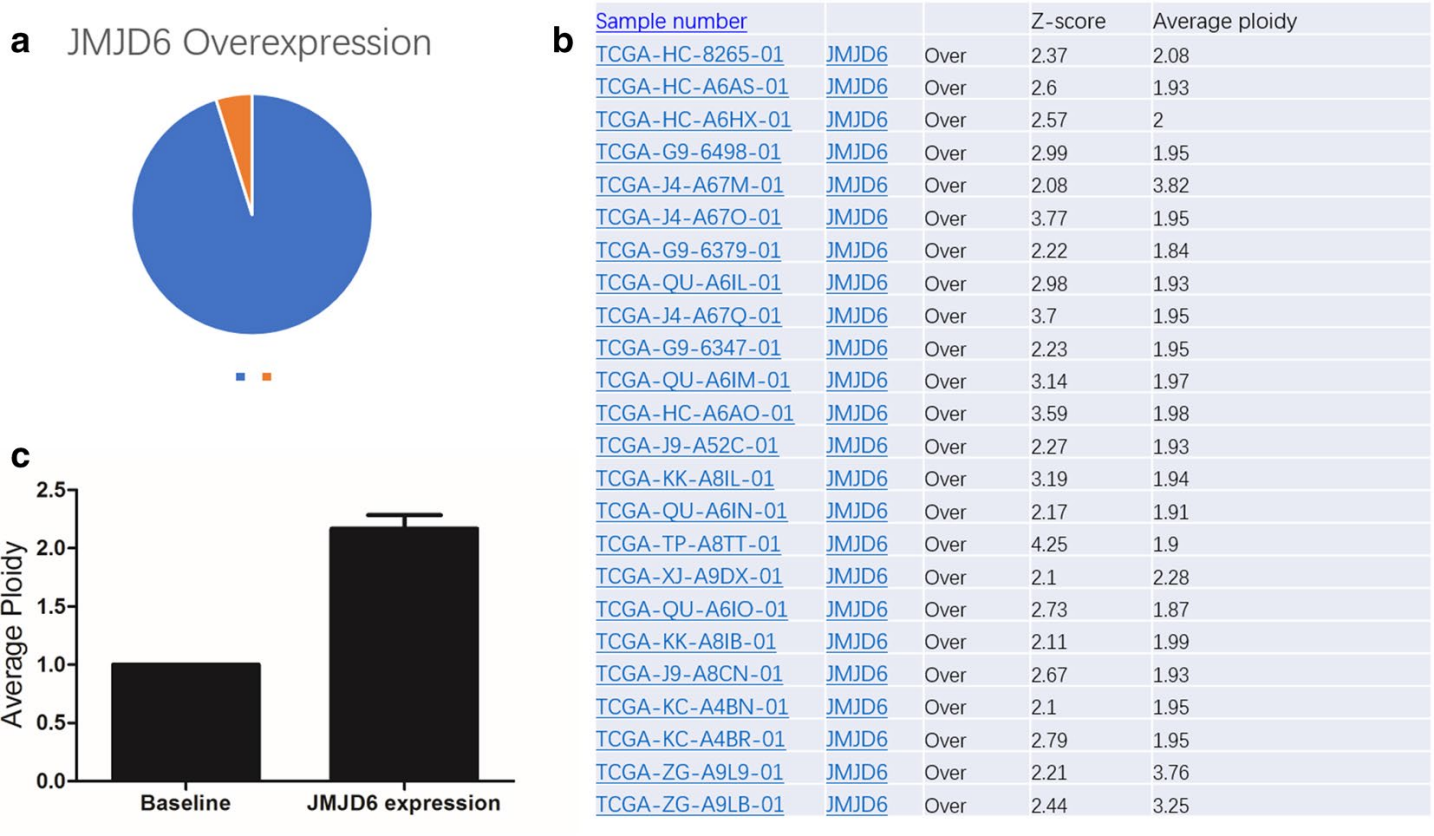
**a** The ratio of JMJD6 overexpression in prostate cancer sample database. **b** The sample number associated with JMJD6 overexpression, Z-score and average ploidy. **c** The statistical result of average ploidy

**Fig. 2:**
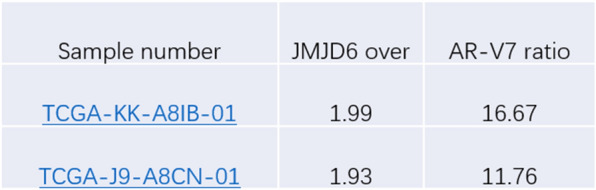
The sample number associated with both JMJD6 and AR-V7 overexpression

According to our bioinformatic analysis from TCGA database, JMJD6 has upregulated expression in prostate cancer patient samples, and shows co-expression and parallel upregulation with AR-V7 (Figs. 1 and 2). Therefore, JMJD6 is considered in relation to prostate cancer development. JMJD6, as a general regulator of the spliceosome, is a key enzyme binding with, hydroxylating and activating its interacting partners/substrates U2AF65-centered spliceosome. Mechanistically, according to previous and our results (data not shown), JMJD6 and U2AF65 appear to co-bind with the pre-mRNAs and co-regulate alternative AR splicing in both JMJD6 enzymatic activity dependent and independent manner. In detail, JMJD6 and U2AF65 seem to preferentially promote exon inclusion based on analysis report on genome-wide mapping of U2AF65 RNA binding sites using CLIP-Seq. K15, K38 or K276 hydroxylation of U2AF65 are deemed to affect alternative splicing and appears independent of JMJD6 enzymatic activity, remaining expecting future investigation [16].

Recent studies specially focus on AR splicing regulation including U2AF65 as one of representatives. Elevated U2AF65 could significantly increase AR-V7 protein expression but no effect on steroidogenesis gene AKR1C3, indicating that AR gene splicing is highly active and plays important role in CRPC. Under ADT conditions, recruitment of RNA splicing factors to the 3′ splicing site of AR-V7 was increased. U2AF65 and ASF/SF2 binding with two kinds of RNA splicing enhancers called intronic splicing enhancer (ISE) and exonic splicing enhancer (ESE) near AR-V7 splicing domain, 3′ss of exon 3B, were identified to play critical roles in AR pre-mRNA splicing to generate AR-V7. The ISE was predicted to bind U2AF65 [10]. According to RNA pull-down assay and conducted MS analysis, ISE was used to identify the potential splicing factors conducting AR-V7 splicing. U2AF65, SAP155, and U2AF35 were screened to be potential candidates. Thailanstatins (TSTs), mainly TST-D, is able to inhibit AR-V7 gene splicing by blocking the interaction between U2AF65 and SAP155, and interfering binding to polypyrimidine tract between the branch point and the 3′ splice site [17].

## Consequences of the hypothesis and discussion

**Fig. 3:**
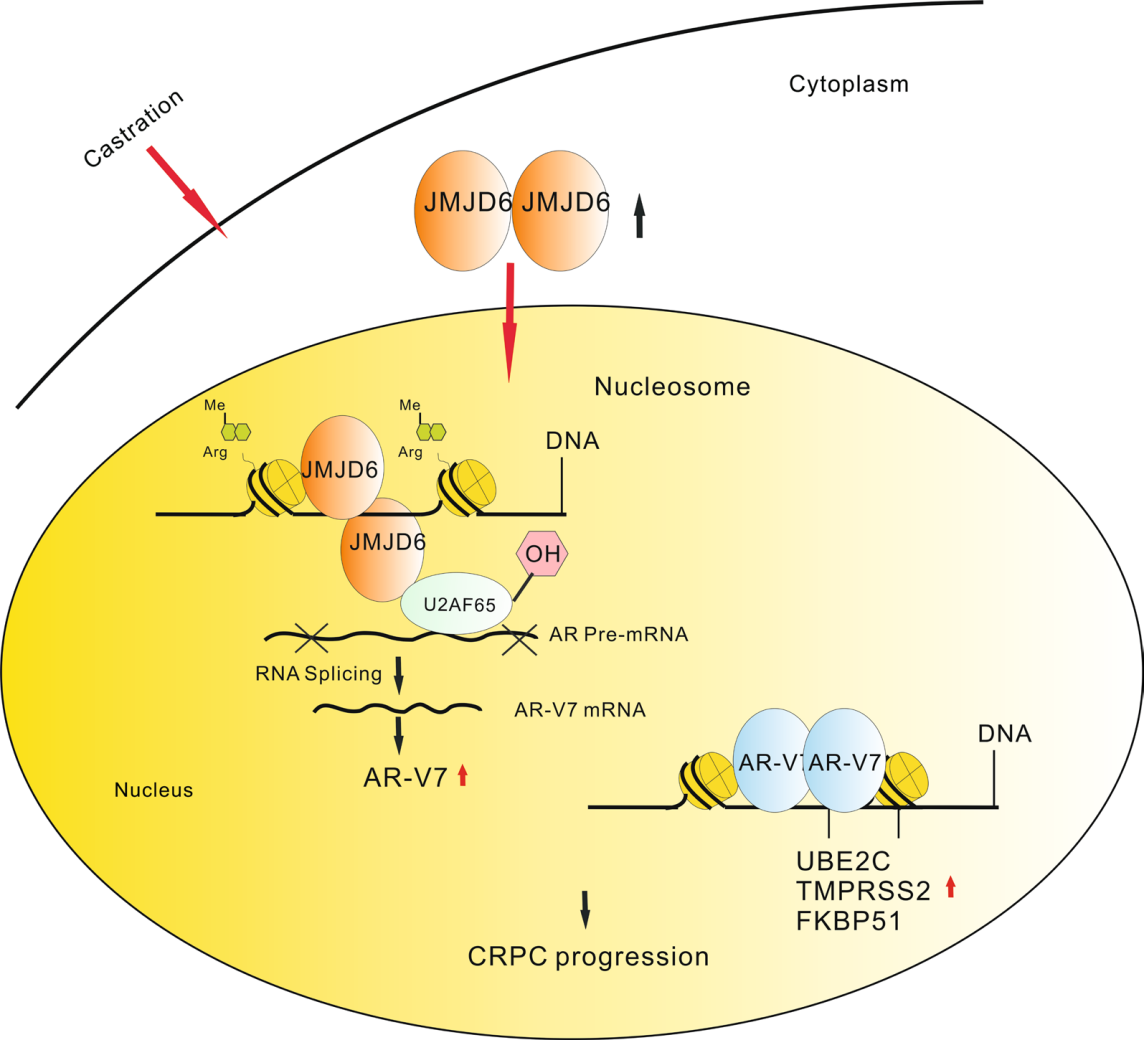
The model elucidating AR mRNA splicing for CRPC progression based on dual-function enzyme JMJD6 and its induced JMJD6/U2AF65/AR-V7 axis

JMJD6 may be a dual enzyme guiding initial AR mRNA transcription via demethylase activity and further AR mRNA splicing to AR-V7 mRNA via hydroxylating U2AF65 (Fig. 3). The role of JMJD6 in the regulation of pre-mRNA splicing via interactions with multiple SR domain and SR-related proteins such as U2AF65, SRSF11, Luc7-like protein 3 and Acinus S, have been described. In fact, multiple splicing factors including U1A, U2AF65, AFS/SF2, hnRNPI, PSF and p54nrb have been demonstrated to play essential roles in RNA splicing under DHT treatment, with no changes in protein levels of these splicing factors observed [10]. Therefore, functional activation of splicing factor members is main factor leading to AR-V7 splicing and providing a strategy to eradicate AR-V7-related CRPC.

According to our hypothesis, activated U2AF65 by JMJD6 plays key roles in regulating AR-V7 splicing. Testing the hypothesis will be challenging. The first topic needed to be addressed is that JMJD6 binds with and catalyzes U2AF65, which further conducts AR-V7 splicing. The complex of JMJD6, U2AF65 and AR mRNA should be verified via IP and RNA-IP assays. Furthermore, suppression and activation of JMJD6 regulates AR-V7 expression by regulation of U2AF65 should be confirmed.

Among JMJD family members, JMJD1A was reported to promote the alternative splicing of AR-V7 by JMJD1A–HNRNPF interaction mechanistically [18–20]. Just like JMJD1A–HNRNPF interaction, JMJD6 and U2AF65 may form a complex to promote AR mRNA transcription under certain conditions such as castration. Furthermore, the splicing factor complexes including JMJD6 and U2AF65 are transient and reassembled in different splicing process. Therefore, JMJD6/U2AF65 induce AR mRNA transcription sometimes, not always.

JMJD6 includes four annotated transcripts or isoforms like JMJD6-1, JMJD6-2, JMJD6-3(JMJD6-Ex5) and JMJD6-4 due to diverse expression of exons. JMJD6-2 located in nucleoplasm and JMJD6-Ex5 in nucleolus and nuclear speckles are the most abundant variants in many human tissues and cell lines [21, 22]. JMJD6-2 and its catalytic inactive mutant JMJD6 AxA decreased exon inclusion in the manner of U2AF65-MS2 independent, suggesting that JMJD6-2 does not involve its hydroxylase activity or its interaction with U2AF65-MS2. JMJD6-Ex5 splicing regulation seemed to be independent on U2AF65 and provided a binding site for hnRNPs, suggesting a regulatory role by hnRNP family members. In accordance with the results from Yi et al. [16], 26% of JMJD6 regulated alternative splicing processes were not co-interacted with U2AF65 and 36% of JMJD6 induced exon skipping events were independent of its enzymatic activity. JMJD6-Ex5 is not involved in splice regulation of the reporter gene via interactions with polyS-domain-containing protein FCP1 and RNA polymerase I transcription factor upstream binding factor UBF [23]. JMJD6 play functions may depend on their separate subnuclear localisation and their almost exclusive protein interactomes. Moreover, JMJD6, previously referred to as phosphatidylserine receptor (PSR) gene, was initially identified as a membrane protein that participates in phagocytosis [24]. JMJD6/PSR regulate phagocytosis of apoptotic cells by diverse signal pathway or molecule including TGF-β, Wnt, CD91 or Rac1 and angiogenesis by VEGF. Furthermore, the structure, localization, and characterization of domains on the JMJD6/PSR that mediate signaling for phagocytosis and cytokine production are currently under investigation. Due to diversity of JMJD6 isoforms and functions, inhibiting JMJD6 as a general regulator of spliceosome simply may not affect homeostatic functions of that gene such as regulation of phagocytosis of apoptotic cells or angiogenesis evidenced in previous reports [14, 25, 26].

Combating AR-V7 is challenging due to lack of targeting drugs and therapy resistance. Some drugs like niclosamide and ONC201 could inhibit AR-V7 signaling [27, 28]. However, there is limited effects in inhibition of AR-V7 expression or related signaling pathway. Regulation of AR-V7 mRNA splicing may be a potential target for combating CRPC. A series of novel drugs, like Onalespib, has been verified to be able to block AR-V7 mRNA splicing [29]. Our viewpoint proposed that targeting JMJD6/U2AF65 pathway may be a new avenue for inhibiting CRPC development. For example, both JMJD6 demethylase and hydrolase inhibitors may be explored to potentially block AR-V7 mRNA splicing. Ultimately, were further study results on the relationship of JMJD6/U2AF65/AR-V7 be explored and found, it might help to reduce potential harm to at-risk CRPC patients. Moreover, it could lead to the development of newer drug targeting JMJD6/U2AF65/AR-V7 axis.

## Data Availability

The datasets used and/or analyzed during the current study are available from the corresponding author on reasonable request.
