# Correction to: The role of JMJD6/U2AF65/AR-V7 axis in castration-resistant prostate cancer progression

**DOI:** 10.1186/s12935-021-02010-x

**Published:** 2021-06-09

**Authors:** Dali Tong

**Affiliations:** Department of Urology, Daping Hospital1, Army Medical University, Chongqing, People’s Republic of China

## Correction to: Cancer Cell Int (2021) 21:45 10.1186/s12935-020-01739-1

Following the publication of the original article [1], the authors have realized that the grant number in the Funding section was written incorrectly.

Essentially, ‘cstc2018jcyjA2133’ was the acceptance number, and the project number (cstc2018jcyjAX0645) should have been written here instead.

Therefore, the text for the Funding section should have been written as follows (the changed text is highlighted in bold):

“This work was supported in part by the Dali Tong Chongqing Science and Technology Bureau (Basic and Frontier Research Project; grant no. **cstc2018jcyjAX0645**) and the Chongqing Municipal Health and Health Committee (Science and Health Joint Medical Research Project; grant no. 2018QNXM041).”

The authors regret their oversight in providing this incorrect information in the Funding section of their paper. They apologize to the readership of the Journal and to the inappropriately credited funding body in question for any inconvenience caused.

## References

[1] Tong D (2021). The role of JMJD6/U2AF65/AR-V7 axis in castration-resistant prostate cancer progression. Cancer Cell Int.

